# Interaction between the NS4B amphipathic helix, AH2, and charged lipid headgroups alters membrane morphology and AH2 oligomeric state — Implications for the Hepatitis C virus life cycle

**DOI:** 10.1016/j.bbamem.2015.04.015

**Published:** 2015-08

**Authors:** Esther L. Ashworth Briggs, Rafael G.B. Gomes, Malaz Elhussein, William Collier, I. Stuart Findlow, Syma Khalid, Chris J. McCormick, Philip T.F. Williamson

**Affiliations:** a Centre for Biological Sciences/Institute for Life Sciences, University of Southampton, Highfield Campus, Southampton SO17 1BJ, UK; b School of Medicine, University of Southampton, Southampton SO16 6YD, UK; c School of Chemistry, University of Southampton, Southampton SO17 1BJ, UK

**Keywords:** POPC, 1-palmitoyl-2-oleoyl-sn-gylcero-3-phosphatidylcholine, POPG, 1-palmitoyl-2-oleoyl-sn-glycero-3-[phospho-rac-(1-glycerol)], ^2^H-POPC, 1-palmitoyl-(d31)-2-oleoyl-sn-glycero-3-phosphatidylcholine, ^2^H-POPG, 1-palmitoyl-(d31)-2-oleoyl-sn-glycero-3-[phospho-rac-(1-glycerol)], Hepatitis C Virus, Membrane remodelling, Solid-state NMR, Membrane biophysics, Molecular dynamics

## Abstract

The non-structural protein 4B (NS4B) from Hepatitis C virus (HCV) plays a pivotal role in the remodelling of the host cell's membranes, required for the formation of the viral replication complex where genome synthesis occurs. NS4B is an integral membrane protein that possesses a number of domains vital for viral replication. Structural and biophysical studies have revealed that one of these, the second amphipathic N-terminal helix (AH2), plays a key role in these remodelling events. However, there is still limited understanding of the mechanism through which AH2 promotes these changes. Here we report on solid-state NMR and molecular dynamics studies that demonstrate that AH2 promotes the clustering of negatively charged lipids within the bilayer, a process that reduces the strain within the bilayer facilitating the remodelling of the lipid bilayer. Furthermore, the presence of negatively charged lipids within the bilayer appears to promote the disassociation of AH2 oligomers, highlighting a potential role for lipid recruitment in regulating NS protein interactions.

## Highlights

• Changes in membrane morphology studied by ^2^H and ^31^P Solid-state NMR.
• Bilayer charge influences the oligomeric state of the amphipathic helix AH2 from NS4B.
• Interaction of AH2 with charged lipid membranes reduces strain within bilayer.
• AH2 from NS4B is involved in membrane remodelling and membranous web formation.
• Lipid bilayer/NS4B interactions may regulate Hepatitis C virus lifecycle.

## Graphical abstract

**Figure:**
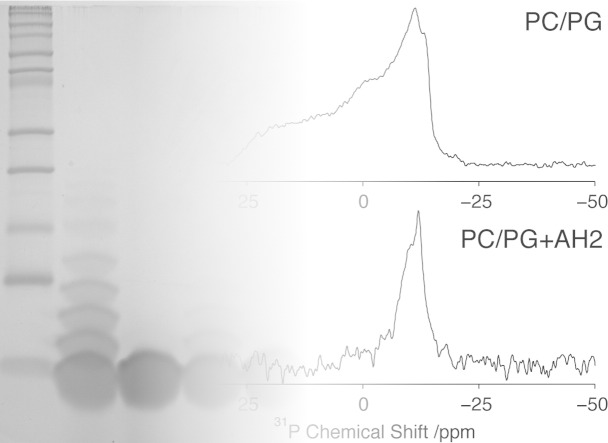

## 1 Introduction

Hepatitis C Virus (HCV) is a major human pathogen infecting an estimated 170 million people globally. HCV, a member of the Flaviviridae family, is a positive sense, single stranded RNA virus with a genome of 9.6 kb containing a single open reading frame (ORF) flanked by 5′- and 3′-untranslated regions [1]. The ORF codes for a single polyprotein of approximately 3000 residues that cellular and viral proteases cleave into four structural proteins (core, E1, E2 and P7) and six non-structural proteins (NS2, NS3, NS4A, NS4B, NS5A and NS5B) [2], [3], [4], [5]. Together with a number of cellular factors, the non-structural proteins contained within the NS3-5B polyprotein precursor facilitate replication of the viral genome within a membrane-associated complex, originally described as a membranous web [6], [7]. Whilst this terminology is still used, more recent electron microscopy studies suggest that the membranous web likely consists of a collection of double and single membrane vesicles derived from the endoplasmic reticulum (ER), with replication restricted to the former structures [8], [9], [10].

**Fig. 1:**
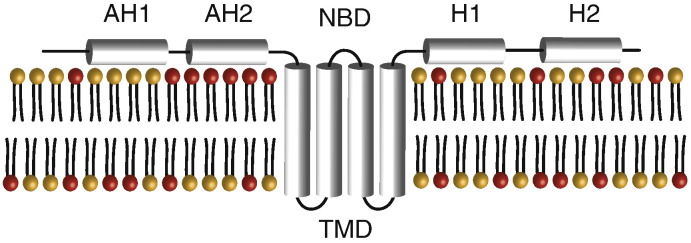
Proposed topology of NS4B within the ER membrane with the relative positioning of the two-amphipathic helices AH1 and AH2, the two C-terminal helices H1 and H2, the four transmembrane domains (TMD) and the location of the nucleotide-binding domain (NBD). During maturation, it is suggested that AH2 may traverse the bilayer, leading to the translocation of AH1 to the opposite face of the membrane [13], [15], [17].

Formation of structures resembling the membranous web can be achieved by expression of the NS3-5B polyprotein in the absence of viral replication [6], and whilst each viral protein contributes to the remodelling activity, NS4B appears to play a major role. NS4B is a 27 kDa integral membrane protein possessing NTPase activity [11], [12]. Its central domain is composed of four transmembrane segments flanked at the N-terminus by two amphipathic helices (AH1 and AH2), and two helical regions (H1 and H2) at the C-terminus [11], [12], [13], [14], [15], [16], [17] (See Fig. 1). Mutations throughout NS4B have been shown to disrupt its ability to cluster itself and other components of the NS3-5B polyprotein into distinct intracellular foci [15], [18], [19], a property linked to membranous web formation. Whilst NS4B-dependent remodelling events partly depend on interactions with both host proteins [20] and other viral NS proteins [21], certain attributes of the second amphipathic helix (AH2) suggest that NS4B also has a direct physical role in driving remodelling events. The first of these is that AH2 experiences a dual topology, lying both on the cytoplasmic surface of the ER membrane, as well as being able to transition across the bilayer to the ER lumen [13], [15], [17], a feature that would in theory allow the protein to drive membrane curvature through a process of wedging. AH2 has also been shown to be a key determinant of NS4B oligomerisation, consistent with the protein driving membrane curvature through a process of scaffolding [15]. Mutations in AH2 that disrupt both these two features block viral replication and affect the appearance of the membranous web/NS protein foci in cells. A final feature of AH2 important for its role in membrane remodelling is that it exhibits specific interactions with lipids present in the bilayer including a range of anionic phosphatidylinositol phosphates [22], lipids whose levels within the bilayer are important for the replication of HCV and many other positive strand RNA viruses [23], [24]. Recruitment/retainment of phosphatidylinositol phosphates and other lipids within the membranous web by NS4B could help modulate bilayer malleability and alter local curvature.

Although the structure of NS4B has yet to be determined, NMR studies of AH2 have revealed that the peptide adopts a monomeric α-helical conformation in detergent micelles [15]. Fluorescence-quenching studies have shown that upon reconstitution into lipid bilayers, the amphipathic helix lies at a shallow location close to the bilayer surface, consistent with at least one of its proposed topologies [22]. Whilst these properties appear to be largely invariant with respect to the lipid bilayer compositions studied to date, the effect of the AH2 domain on lipid bilayers and its potential role in membrane remodelling remains unclear. The addition of AH2 to vesicle suspensions has been demonstrated to alter a number of bilayer properties, including perturbing the phase transition properties and triggering vesicle aggregation [25], [26]. The presence of AH2 has also been shown to lead to vesicle leakage, the rates of which show a strong lipid dependence with negatively charged bilayers significantly less perturbed than their neutral counterparts [22]. Complementary ^31^P-NMR studies of lipid bilayers in the presence of AH2 have been conducted and revealed no significant changes in the bilayer structure and integrity, however these studies have focussed primarily on neutral bilayers, largely ignoring the presence of anionic lipids such as those implicated in viral replication [22].

Here we report on a series of studies that investigate how the surface charge of the lipid bilayer influences the oligomeric state of AH2, and how these interactions modulate lipid bilayer morphology, to determine how these effects may influence membranous web formation. Coarse grain molecular dynamics studies have revealed that AH2 association with the surface of negatively charged lipid vesicles containing either the anionic lipids phosphatidylinositol-4,5-bisphosphate (PIP2) or phosphatidylglycerol result in the clustering of anionic lipids around the peptide. Chemical cross-linking studies show that the association of AH2 with the negatively charged lipid bilayer results in the disassociation of AH2 oligomers into smaller species when compared with neutral bilayers. The recruitment of the negatively charged lipids to AH2 and its disassociation into smaller oligomeric species results in a notable reduction in bilayer stress as evidenced by the enhanced bilayer deformation observed in the ^31^P NMR studies. These findings highlight the important role that the recruitment of negatively charged lipids by the AH2 domain of NS4B to the reaction centre play in determining both the oligomeric state of NS4B and the underlying remodelling of the ER to form membranous webs.

## 2 Materials and methods

### 2.1 Reagents

A peptide composed of the sequence NH_3_^+^-WPKVEQFWARHMWNFISGIQYLAG-COO^−^ was prepared using conventional FMOC chemistry by PeptideSynthetics (Fareham, UK). The peptide was used as supplied at > 70% purity and gave rise to a single species at 2965 Da when analysed by mass spectrometry. The peptide corresponds to the second amphipathic helix in the non-structural protein NS4B (residues 1758 to 1781 of the polyprotein from the JFH1 genotype 2a, residues 43 to 66 of NS4B). All lipids used in these studies were purchased from Avanti Polar Lipids (Alabaster, USA) and used without further purification. All other reagents were purchased from Sigma, unless stated otherwise.

### 2.2 Chemical cross-linking with TCA precipitation

To reconstitute the AH2 peptide into POPC and POPC/POPG (ratio 2:1) vesicles the lipids (10 mg/ml stock solutions in methanol) were mixed with 40 μg of peptide (2 mg/ml stock solution in methanol) at a L/P ratio of 100:1. The samples were dried under vacuum overnight to remove the methanol. The resulting lipid-peptide film was re-suspended in 100 μl of 5 mM sodium phosphate buffer, pH 7.4. The rehydrated samples were then sonicated to clarity for 20 min, using a bath sonicator (Ultrawave U100) to form small unilamellar vesicles (SUVs). The use of SUVs increases the bilayer curvature compared to the multilamellar vesicles (MLVs) employed in NMR studies but allows the effect of surface charge on peptide aggregation to be determined whilst reducing the possibility of cross-linking between peptides bound to adjacent bilayers which may potentially occur in MLVs. Chemical cross-linking was performed using DSS (Disuccinimidyl suberate, Sigma Aldrich) at a ratio of 18 μg DSS (stock solution of 90 μg/μl in DMSO) per μg of reconstituted peptide and the reaction was allowed to proceed for 1 h at room temperature as described by Marius et al. 2012 [27].

The protein was subsequently precipitated through the addition of 100% w/v stock solution of TCA at a ratio of 1 to 4 with respect to the sample volume. The samples were incubated on ice for 10 min and pelleted at 13,000 rpm in a bench top centrifuge. The supernatant was removed and the pellet was washed twice with 200 μL of ice-cold acetone. The pellet was then dried at 95 °C, for 10 min, to drive off any remaining acetone and subsequently dissolved in 20 μL of loading buffer and boiled for 10 min at 95 °C. The samples were analysed by SDS-PAGE (16% tricine gel) and stained with InstantBlue (Expedeon).

### 2.3 Preparation of samples for solid-state NMR

Lipid vesicles in the absence and presence of AH2 were prepared by dissolving POPC or a mixture of POPC/POPG at a molar ratio of 2:1 in chloroform/methanol. Typically, 10 mg of total phospholipid were prepared for each sample. For samples containing AH2, the peptide was added from a stock solution in methanol to achieve the desired lipid to protein ratio. Samples were subsequently dried under high vacuum overnight to remove any residual solvent and subsequently rehydrated (30% w/v) in low salt buffer (10 mM Tris, 1 mM EDTA, 10 mM NaCl; pH 7.4). The samples were then subjected to five cycles of freeze thawing resulting in a homogeneous suspension of multi-lamellar vesicles (MLVs). For deuterium NMR the appropriate lipid was replaced with its deuterated counterpart.

### 2.4 Static ^31^P-NMR

Static ^31^P-NMR spectra were acquired at 161 MHz on a Chemagnetics Infinity 400 MHz NMR spectrometer using a Chemagnetics double-resonance 4 mm magic-angle spinning (MAS) probe without spinning. Static measurements were performed using a Hahn-echo pulse sequence. Spectra were excited with a 90° pulse of 4.5 μsec, with an inter-pulse spacing of 30 μsec. Continuous wave (CW) proton decoupling (50 kHz) was applied during acquisition, with a 2.5-second recycle delay, to ensure the sample was not heated. Typically, 512 scans were accumulated for each temperature step. Chemical shifts were externally referenced to H_3_PO_4_ (85%). Prior to Fourier Transform, data were left shifted to the top of the echo, zero filled to 4096 points and 75 Hz line broadening applied.

### 2.5 Deuterium NMR

Deuterium NMR spectra were acquired at 61.5 MHz on a Chemagnetics Infinity 400 NMR spectrometer, using a Chemagnetics double-resonance 4 mm MAS probe without spinning. Static measurements were made using a quadrupolar echo pulse sequence with a 90°-pulse of 4 μsec, an inter-pulse delay of 50 μsec and a recycle time of 500 msec. Typically, 32,768 acquisitions were accumulated for each temperature step. Prior to Fourier Transform the data was left shifted to the top of the echo, zero filled to 4096 points, and 100 Hz linebroadening was applied.

The spectra were Depaked using a weighted Fast Fourier Transform algorithm [28] using custom written routines in Matlab (Mathworks Inc.). The observed quadrupolar splittings (*ν*_*Q*_^*i*^) were used to calculate the order parameters (*S*_*CD*_^*i*^) directly as described previously:

$$S_{CD}^{i}=\frac{4}{3}.\frac{\Delta \nu_{Q}}{\Delta \nu_{Q}^{\mathrm{Static}}}$$

where *ν*_*Q*_^*Static*^ is the static quadrupolar coupling constant (*e^2^qQ*/*h*), which is 167 kHz for a paraffinic C-D bond [29]. The order parameter profiles were constructed based on previously published assignments [30].

### 2.6 Coarse-grain models

All CG simulations were performed using GROMACS 4.5.5 (www.gromacs.org) [31], [32], [33] with the MARTINI CG 2.0 force field [34]. The parameters for the PIP2 lipids were as described by Stansfeld et al. 2009 [35]. All simulations involved self-assembly of a lipid bilayer from a random configuration of lipids, ions and water as described in [36], [37], [38]. Varying numbers of peptides, 3, 5 or 10 (PDB code = 2JXF) were then added to the system in the bulk water region, details of the simulation systems are given in Table 1. The integrity of the HCV helix was retained through the implementation of an elastic network model.

### 2.7 Simulation parameters

For all CG simulations, Lennard–Jones interactions were smoothly shifted to zero between 9 Å and 12 Å, and electrostatics were smoothly shifted to zero between 0 Å and 12 Å, with a relative dielectric constant of 20 used for explicit screening. The nonbonded neighbour list was updated every 10 steps. All simulations were performed at constant temperature, pressure, and number of particles. The temperatures of the protein, POPC, POPG, PIP2, and solvent were each coupled separately using the Berendsen algorithm [39] at 300 K, with a coupling constant τT = 1 ps. The system pressure was anisotropically coupled using the Berendsen algorithm at 1 bar with a coupling constant τP = 1 ps and a compressibility of 5 × 10^−6^ bar^−1^. The time step for integration was 10 fs. Analyses of the CG simulations were performed using GROMACS tools and locally written code and visualization used VMD [40].

## 3 Results

### 3.1 Solid state phosphorus NMR

**Fig. 2:**
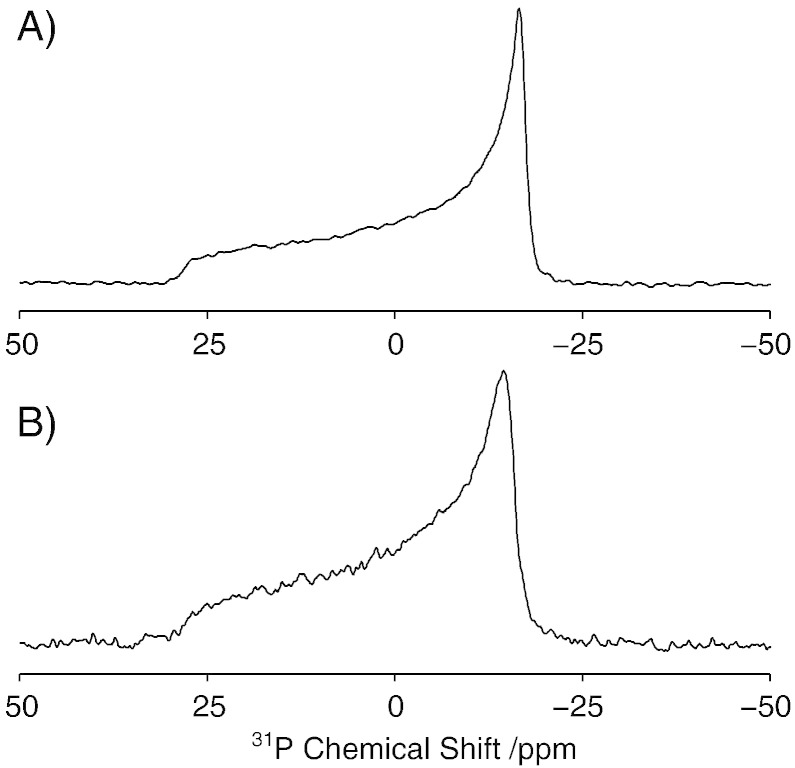
Effect on bilayer morphology of the addition of AH2 to neutral vesicles. Pure POPC vesicles (A) and POPC vesicles with AH2 present at a lipid/protein ratio of 100:1 (B). Data acquired at 25 °C.

To assess how AH2 affects integrity of the lipid bilayer, ^31^P static NMR spectra were recorded in the presence and absence of AH2. The static ^31^P spectra of multilamellar vesicles in the absence and presence of AH2, at a lipid/protein ratio of 100:1, are shown in Fig. 2. In the absence of AH2, the static ^31^P spectra of POPC multilamellar vesicles exhibit a classical axially symmetric powder pattern, characterised by a chemical shielding anisotropy of 30.8 ppm (Fig. 2A). A similar axially symmetric powder pattern is observed upon the addition of AH2 to POPC multilamellar vesicles, indicating that no significant disruption of these neutral bilayers occurs in the presence of AH2 (Fig. 2B). Although a slight broadening of the powder pattern is observed, consistent with a small change in T_2_ resulting from small changes in headgroup dynamics, there is no significant change in the chemical shielding anisotropy in the presence of AH2 , suggesting that the lipids are not immobilized. These findings support those by Palomares–Jerez et al. [22], who reported that bilayer integrity remained intact in neutral lipid bilayers composed of egg phosphatidylcholine and egg sphingomyelin that were studied at lower lipid/protein ratios.

**Fig. 3:**
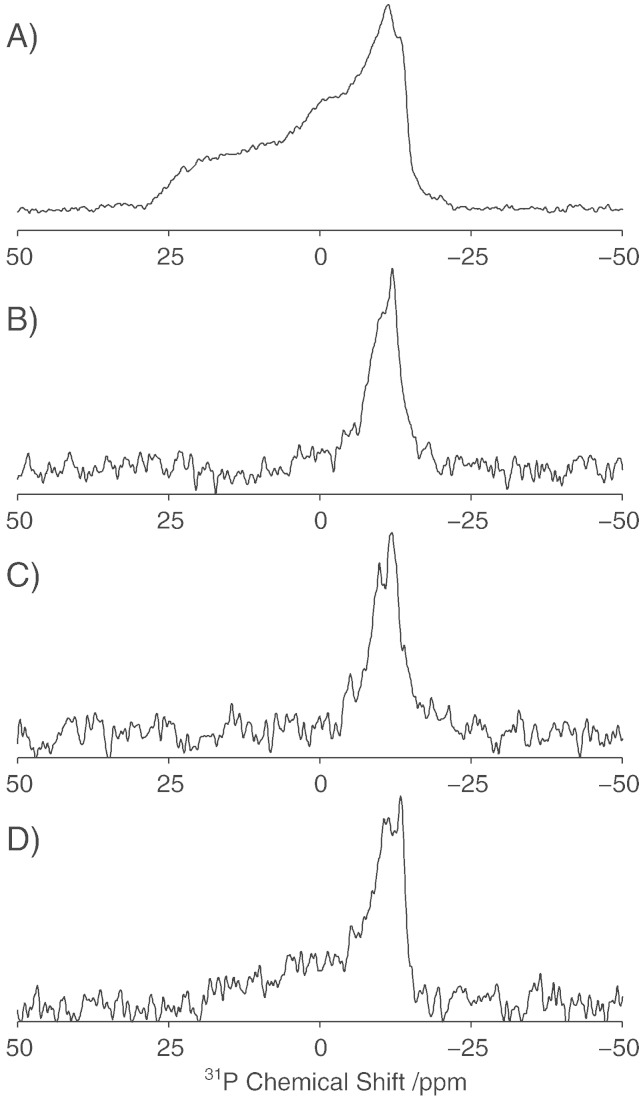
Effect on bilayer morphology of the addition of AH2 to negatively charged vesicles composed of POPC/POPG (2:1). Pure POPC/POPG vesicles (A), with AH2 added at a 50:1 lipid/protein ratio (B), 100:1 lipid/protein ratio (C) and 200:1 lipid/protein ratio (D). Data acquired at 25 °C.

To investigate the effect of bilayer charge on the interaction of AH2 with the lipid bilayer and to assess its effect on bilayer integrity, ^31^P static NMR spectra were recorded from multilamellar vesicles composed of POPC and POPG at a molar ratio of 2:1 (Fig. 3). In the absence of AH2, the lineshapes observed are consistent with the superimposition of the POPC and POPG lineshape, with the π/2 edges of the powder lineshapes at −11.3 ppm and −13.3 ppm for POPG and POPC, respectively (Fig. 3A). Samples of POPC/POPG vesicles with AH2 were prepared at lipid/protein ratios of 50:1, 100:1 and 200:1 (Fig. 3B, C and D, respectively). In contrast to the pure POPC/POPG vesicles, the presence of AH2 appears to disrupt the bilayer, evidenced by the disappearance of the classical axially symmetric powder pattern, resulting in resonances close to the π/2 edges of the powder lineshapes. The positions of these peaks show a downfield shift with increasing peptide concentration, moving from −11.3 ppm and −13.3 ppm at a lipid to protein ratio of 200:1 (Fig. 3D), to −9.9 ppm and −11.9 ppm at a lipid to protein of ratio of 50:1 (Fig. 3B). We note that the sensitivity in the POPC/POPG spectra in the presence of AH2 are typically less than observed for the POPC samples. To assess if this was due changes in relaxation resulting from changes in dynamics in the sample extensive relaxation studies were performed (data not shown). No significant differences were observed in T_1_ or T_2_ relaxation rates that can account for the reduced spectral intensity. Magic-angle spinning ^31^P-NMR spectra were also acquired (data not shown), to assess if the changes observed in the chemical shielding anisotropy arose from charge compensation on the surface of the bilayer upon binding of AH2 [41]. In keeping with earlier findings, no perturbations were observed in the isotropic chemical shifts of either POPC or POPG [22]. This suggests that the small differences in chemical shielding anisotropy detected in these measurements reflect a slight increase in headgroup mobility in the presence of AH2. Nevertheless, the disappearance of the downfield intensity in the powder lineshape is indicative of a change in the orientational distribution of the lipids within the sample, and is consistent with a deformation of the multilamellar vesicles in the sample. This deformation is due to the lipids aligning perpendicular to the magnetic field, caused by their negative diamagnetic anisotropy, and is inconsistent with the solubilisation of the bilayer [42], [43], [44]. This observation suggests that AH2 has reduced the energy required for bilayer deformation, as has previously been observed for a number of other surfactant-like proteins, including antimicrobial peptides such as melittin, and antifreeze proteins [45], [46], [47], [48].

### 3.2 Deuterium NMR

**Fig. 4:**
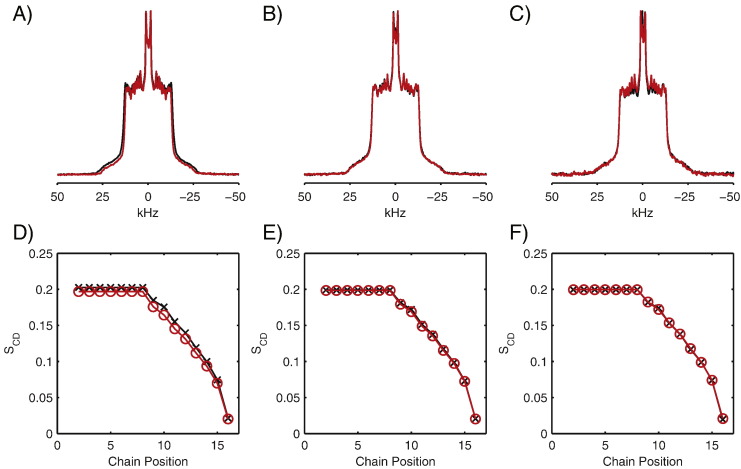
Deuterium NMR spectra of vesicles in the absence (black) and presence of AH2 at a lipid/protein ratio of 100:1 (red). Pure d_31_-POPC vesicles (A), d_31_-POPC/POPG (2:1) (B) and POPC/d_31_-POPG (2:1) (C). Corresponding order parameter profiles for d_31_-POPC vesicles (D), d_31_-POPC/POPG (2:1) (E) and POPC/d_31_-POPG (2:1) (F) in the presence (red) and absence (black) of AH2. Data acquired at 25 °C.

To assess the effect of AH2 on the phase behaviour of the lipid chains and their mobility within the bilayer, deuterium NMR spectra were recorded with each of the individual lipids deuterated on the palmitoyl chain (Fig. 4). The deuterium spectrum of POPC in the absence of AH2 exhibits a classical powder lineshape with the characteristic Pake pattern, with resolvable splittings corresponding to the 9 CD_2_ groups closest to the methyl group of the acyl chain (Fig. 4A). Upon addition of AH2 at a lipid to protein ratio of 100:1, a slight reduction in the quadrupolar splittings is observed. Analysis of the order parameter profile (Fig. 4D) shows a small (2.8%) but reproducible reduction in the order parameter (S_CD_) along the length of the POPC acyl chain, consistent with AH2 increasing the mobility within the lipid bilayer, mirroring the effects observed in the ^31^P spectra.

To test if AH2 interacts specifically with POPC or POPG in mixed POPC/POPG bilayers, deuterium spectra were acquired for samples labelled with either d_31_-POPC or d_31_-POPG. In contrast to pure d_31_-POPC bilayers, the addition of AH2 to POPC/POPG bilayers resulted in no significant perturbations to the deuterium spectra of d_31_-POPC or d_31_-POPG (Fig. 4B and C, respectively). Accordingly, analysis of the order parameter profiles (Fig. 4E and F) revealed no significant changes in the mobility of the lipid chains through the addition of AH2.

### 3.3 Effect of bilayer composition on oligomer formation

**Fig. 5:**
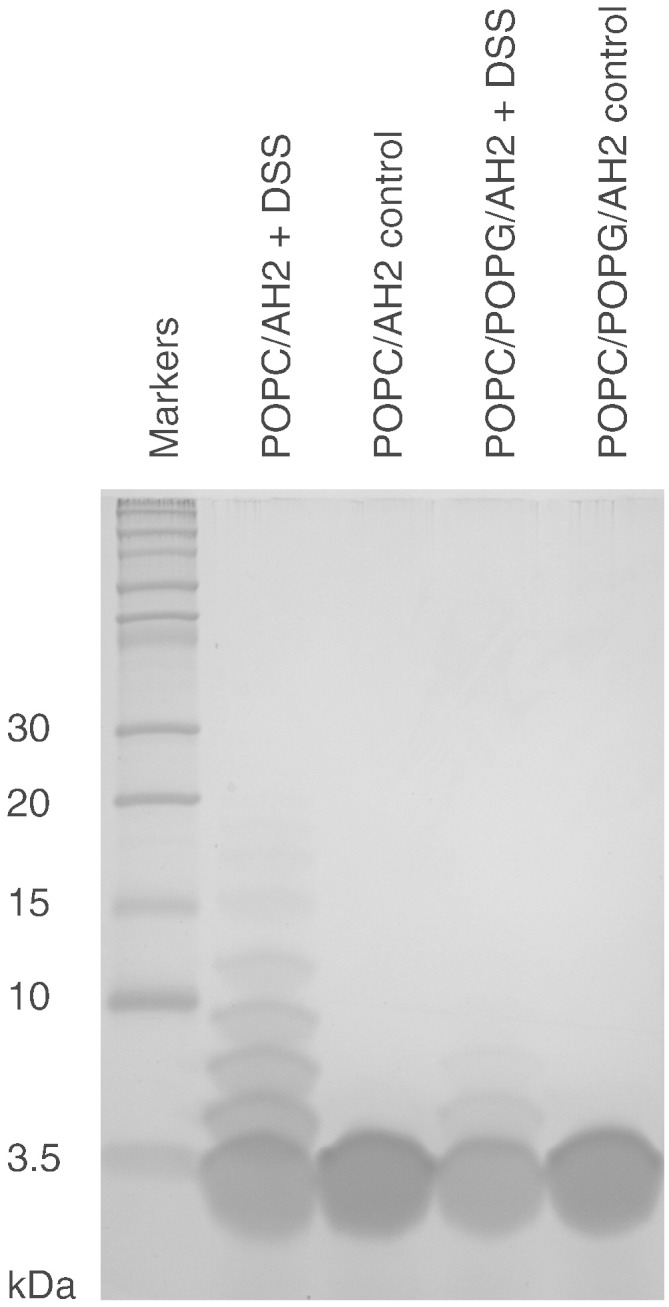
SDS-PAGE of chemically cross-linked AH2 in POPC and POPC/POPG (molar ratio 2:1) vesicles, at a lipid to protein ratio of 100:1. Cross-linking was carried out using DSS, followed by TCA precipitation for removal of lipids. Samples were run on a 16% tricine SDS-PAGE gel, and visualised using InstantBlue stain. Uncross-linked samples show a single band at the expected size with a molecular weight between 2 and 3.5 kDa, corresponding to monomeric AH2 peptide.

The above data highlights that bilayer composition, in particular its charge, appears to play an important role in understanding how AH2 modulates local bilayer morphology. However, it is unclear if alterations in bilayer morphology are brought about by changes in the properties of AH2. A number of studies have suggested that membranous web formation may be brought about by the formation of higher order oligomers of NS4B, which have been proposed to form through interactions between multiple AH2 helices [26]. To address whether bilayer composition influences the oligomeric state of AH2, we have undertaken a series of cross-linking experiments, with AH2 in the presence of neutral lipid vesicles composed of POPC and negatively charged lipid vesicles composed of POPC and POPG (Fig. 5). In the absence of the membrane permeable amine cross-linker DSS, AH2 runs as a monomeric species on the denaturing SDS-PAGE gel, close to its predicted molecular weight of 2965 Da. In neutral bilayers composed of POPC, the presence of the cross-linker results in the formation of a ‘ladder’, indicating the presence of higher oligomeric species within the bilayer, with hexameric complexes readily observable. In contrast, in the presence of negatively charged lipids the degree of cross-linking is significantly curtailed with a trimeric complex being the largest readily observed.

### 3.4 Coarse-grained molecular dynamics (CGMD) studies of the interaction of AH2 with lipid bilayers

**Fig. 6:**
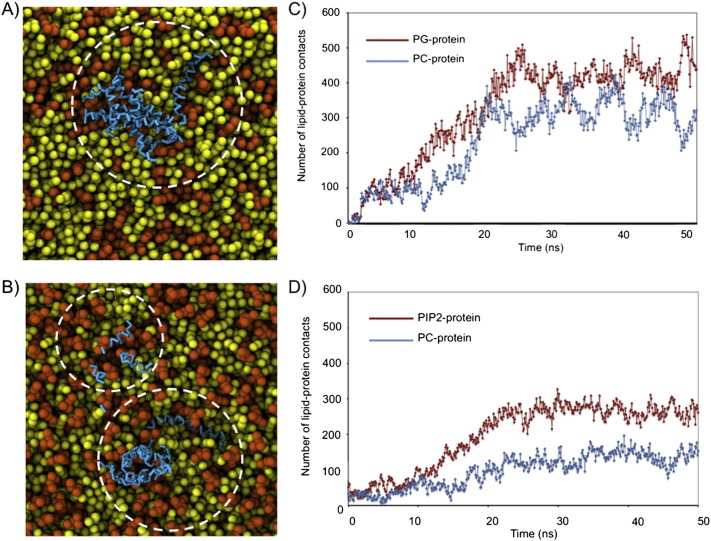
Snapshot of a coarse grain molecular dynamics simulation of 10 AH2 interacting with a POPC/POPG (A) and POPC/PIP2 (B). POPC lipids (yellow) POPG/PIP2 (red). Comparison of number of contacts between POPC and POPG (C) and between POPC and PIP2 (D).

In all simulations containing anionic lipids, the AH2 peptides were observed to diffuse from the bulk water towards the bilayer such that they formed surface-interactions with the model membrane. We did not observe peptide insertion into the hydrophobic core region of the lipid bilayer in any of our simulations. A typical scenario is described below for a system containing 10  peptides with the bilayer composed of a 3:1 ratio of POPC:POPG phospholipids. After only 3 ns of simulation, a single peptide became associated with the surface of the membrane. It quickly adopted an orientation parallel to the plane of the membrane. Higher oligomers, a tetramer, trimer and dimer begin to form in solution after ~ 4 ns with the tetramer becoming surface-associated to the membrane by ~ 7 ns. After ~ 22 ns, the dimer is also surface-associated and aggregates with the already surface-associated tetramer forming a hexameric structure (Fig. 6A). During this time, the trimer becomes surface-associated with the other leaflet of the lipid bilayer. Thus none of the peptides remain detached from the membrane after about ~ 22 ns. Interestingly, the aggregates do not appear to be ordered in anyway, with the peptides arranging in random orientations. Analysis of lipid-protein interactions (where interaction is defined as r ≤ 6 Å) revealed a marked preference for PG lipids over PC lipids (Fig. 6B). For example when all ten peptides are surface associated, there are ~ 450–500 lipid–protein contacts (counting all the lipid and protein particles) with PG lipids but only ~ 400–420 with PC lipids, despite there being a 3:1 excess of the latter. When only two peptides are surface associated then, the number of contacts is ~ 50 for PG lipids and < 10 for PC lipids.

Simulations of the AH2 peptides in 2:1 PC:PIP2 lipid bilayers were also performed as previous studies have revealed a strong association between AH2 and PIP2 [22]. Simulations again revealed that peptide aggregation and bilayer surface association occurred within the first 10 ns primarily through positively charged residues towards the N-terminus of the peptide (Fig. 6B). Analysis of the lipid protein interactions again revealed a stronger preference for the PIP2 lipid than PC with ~ 280 lipid–protein contacts with PIP2 but only ~ 120 with PC lipids, despite a 2:1 excess of the latter (Fig. 6D). The binding of the AH2 to both POPC/POPG and POPC/PIP2 bilayers suggests that binding is mediated by the surface charge on the bilayer, rather than interacting with a specific binding site on AH2. Binding of AH2 to the bilayer surface does however result in the clustering, in both cases, of the negatively charged lipids around the peptide (Fig. 6A and C), a property that could contribute to the deformation of the bilayer necessary for membrane remodelling.

In contrast to these findings, CGMD simulations of AH2 in the presence of neutral POPC bilayers revealed a significantly lower affinity for the bilayer. Although association with the bilayer occurred in these simulations, it was mediated by a small number of AH2 molecules, with aggregation occurring extensively in the aqueous phase and largely through protein/protein interactions (See Supplementary Fig. 1). We refrain from making direct comparison between the aggregation observed in CGMD simulations and cross-linking studies as the concentrations of peptides and lipids differ significantly between the two systems, whilst the limited number and length of simulations precludes an accurate determination of the statistical distribution of the oligomers. The CGMD simulations do however suggest that in the absence of anionic lipids, protein/protein rather than protein/lipid contacts are favoured, which may account for the reduction in size of oligomers observed in cross-linking studies in the presence of anionic lipids.

## 4 Discussion

Our findings reveal that upon reconstitution into neutral lipid bilayers, the AH2 domain from NS4B has a propensity to form higher oligomeric species, which, although slightly increasing the mobility within the centre of the bilayer, appear to have little effect on the integrity of the bilayer. The presence of the anionic lipid POPG within the bilayer reduces the size of the AH2 oligomers forming, as seen in our cross-linking studies. This reduction in oligomeric state is accompanied by significant changes in bilayer properties. Although the order within the headgroup and chain regions for both POPC and POPG remain constant, the bilayers appear to have a reduced rigidity, as evidenced by the bilayer's ability to adopt an aligned phase in the magnetic field, which is seen in the ^31^P static NMR spectra. Molecular dynamics studies suggest that this, in part, may be attributable to the preferred interaction of AH2 with negatively charged lipids within the bilayer, which appears to drive the clustering of the lipids, potentially driving the deformation of the bilayer.

Viral replication has been linked to the need for activation or recruitment of PI4 kinase to the membranous web, although there is some controversy as to whether this is to drive phosphorylation of phosphatidylinositol or NS5A [49]. Nonetheless, the presence of the anionic lipid PI4P at the sites of viral genome replication would be expected to influence the overall charge of the bilayer. It was therefore unexpected that the presence of the anionic lipid POPG in vesicles, designed to mimic the change in charge experienced as NS proteins move from the site of synthesis (ER) to the membranous web, reduced AH2 oligomerization. One explanation may be that it is not charge alone that dictates AH2 behaviour, and that specific interactions of AH2 with the PI4P head group modulate its activity so as to maintain an oligomeric state. In this way, it is possible that AH2 has adapted to overcome the problems that head group acidification through PI4 kinase activity would otherwise bring about. However, the fact that a reduction in AH2 oligomeric status coincides with the peptide's ability to enable lipid bilayer deformation, a property likely to be desirable for membrane remodelling events, suggests that an alternative explanation is likely. As molecular dynamics simulations of both POPG and PIP2 exhibit similar AH2 surface association and clustering of anionic lipids, our preferred model is one where the lipid head group composition regulates the activity of AH2. We envisage that AH2 oligomerisation is essential during early stages of membranous web formation, perhaps contributing to the formation of a higher ordered structure during polyprotein maturation, which serves as a scaffold for initial remodelling events. In later stages, enrichment of the membrane with anionic lipids through the action of PI kinases and/or their clustering and recruitment of anionic lipids around AH2 triggers the disassociation of AH2 resulting in a weakening of the bilayer's tensile strength, facilitating a remodelling event driven by other NS proteins such as NS5A.

## Figures and Tables

**Table 1:** Summary of the coarse grain molecular dynamics simulations performed and summary of the oligomeric states formed.

Simulations (3 independent runs of each)	Number of peptides	Membrane composition	Length of simulation /ns	Multimers formed
PG_HCV3	3	3:1 POPC:POPG	50	2
PG_HCV5	5	3:1 POPC:POPG	50	2, 3, 5
PG_HCV10	10	3:1 POPC:POPG	50	2, 3, 4, 6
PIP2_HCV3	3	2:1 POPC:PIP2	50	2, 3
PIP2_HCV5	5	2:1 POPC:PIP2	50	2, 3, 4
PIP2_HCV10	10	2:1 POPC:PIP2	50	3, 4, 5, 8
PC_HCV	3	100% POPC	50	2, 3
PC_HCV	5	100% POPC	50	3, 5
PC-HCV	10	100% POPC	50	2, 6, 7, 10
