# Supplementary material for: Interaction between the NS4B amphipathic helix, AH2, and charged lipid headgroups alters membrane morphology and AH2 oligomeric state — Implications for the Hepatitis C virus life cycle
Source: Biochim Biophys Acta. 2015 Aug;1848(8):1671–7. doi: 10.1016/j.bbamem.2015.04.015 (PMC4768108; doi:10.1016/j.bbamem.2015.04.015)
Supplement: Supplementary file 1 — Supplementary material 1. [file mmc1.pdf]

Supplementary Information

Interaction between the NS4B amphipathic helix, AH2, and charged lipid headgroups alters membrane morphology and AH2 oligomeric state - implications for the Hepatitis C Virus life cycle.

Esther L. Ashworth Briggs[1], Rafael Gomes[1,2], Malaz Elhussein[3], William Collier[1], I. Stuart Findlow[1], Syma Khalid[3], Chris J. McCormick[2*], Philip T.F. Williamson[1*]

[1]Centre for Biological Sciences/Institute for Life Sciences, University of Southampton, Highfield Campus, Southampton, SO17 1BJ, United Kingdom. [2]School of Medicine, University of Southampton, Southampton, SO16 6YD, United Kingdom. [3]School of Chemistry, University of Southampton, Southampton, SO17 1BJ, United Kingdom.

Corresponding authors:

P.T.Williamson@soton.ac.uk

C.J.McCormick@soton.ac.uk

Figures

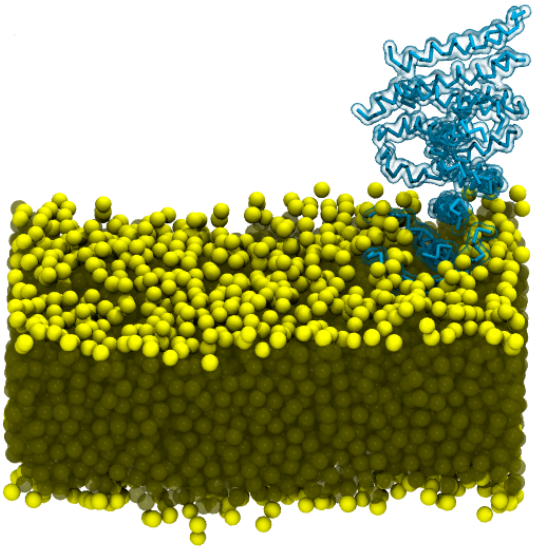

Supplementary Figure 1. Representative snapshot of an AH2 oligomer interacting with the surface of a POPC bilayer. Interactions are mediated by two AH2 molecules, with the oligomeric structure being formed primarily from protein/protein interactions.
